# Competing Mortality Redefines the Net Benefit of Additional Surgery After Endoscopic Resection for T1 Colorectal Cancer in Older Adults

**DOI:** 10.1111/den.70222

**Published:** 2026-07-04

**Authors:** Katsuro Ichimasa, Shin‐ei Kudo, Yuta Kouyama, Taishi Okumura, Yasuharu Maeda, Takemasa Hayashi, Khay Guan Yeoh, Hideyuki Miyachi, Masashi Misawa

**Affiliations:** ^1^ Digestive Disease Center Showa Medical University Northern Yokohama Hospital Yokohama Kanagawa Japan; ^2^ Department of Medicine, Yong Loo Lin School of Medicine National University of Singapore Singapore Singapore; ^3^ Department of Gastroenterology and Hepatology National University Hospital Singapore Singapore; ^4^ Department of Gastroenterology and Hepatology Kochi Medical School, Kochi University Kochi Japan

**Keywords:** aging, colectomy, colorectal neoplasm, lymphatic metastasis, mortality

## Abstract

The oncologic benefit of additional surgery after endoscopic resection (ER) for the treatment of T1 colorectal cancer (CRC) remains uncertain in older adults, because competing causes of mortality may attenuate the gain in survival. The proportion of patients aged ≥ 80 years has increased steadily, reflecting population aging. For patients with high‐risk T1 CRC, the aim of additional bowel resection is to remove occult lymph node metastasis and reduce the risk of recurrence, and long‐term studies have shown improvements in T1 CRC‐related outcomes. However, age modifies the magnitude of this benefit. Cohort studies of high‐risk T1 CRC have shown only small differences in 5‐year cancer‐specific survival between patients who underwent additional surgery and those who did not. Moreover, most deaths in the nonsurgical group were attributable to causes other than cancer. Data from meta‐analyses have further suggested that the survival advantage associated with surgery becomes evident only after 10 years, indicating a substantial delay in its benefits. In contrast, the incidences of perioperative morbidity and short‐term mortality increase with age and have immediate effects on prognosis. These findings indicate that the net survival benefit of additional surgery in older patients depends on the balance between the delayed oncologic benefit and the immediate treatment‐related risks. Thus, although surgery remains appropriate for selected fit individuals, clinicians should consider the pathologic risk, frailty, comorbidity burden, and competing mortality of individual older patients with pT1 CRC in their decision‐making to optimize outcomes.

## 1 Introduction: The Emerging Clinical Dilemma

**FIGURE 1:**
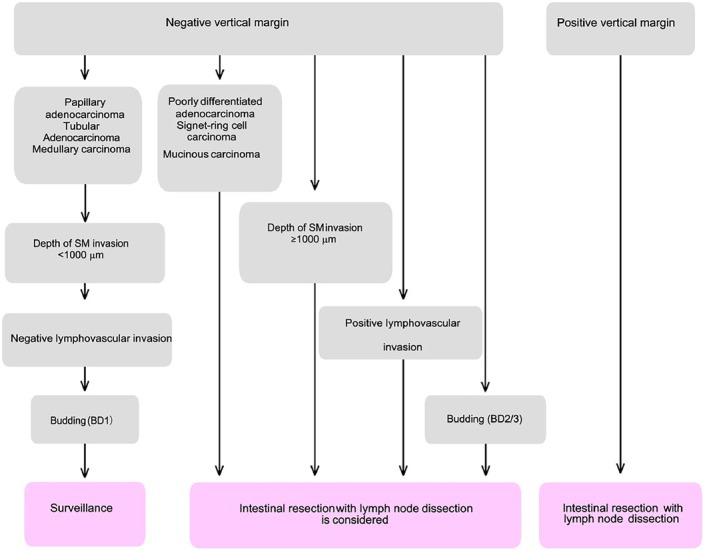
Treatment strategies for pT1 colorectal cancer after endoscopic resection based on the Japanese Society for Cancer of the Colon and Rectum (JSCCR) guidelines [5]. SM, submucosal.

The number of older adults who are diagnosed with pT1 colorectal cancer (CRC) is increasing steadily in parallel with global population aging [1, 2]. In our institutional cohort of T1 CRC patients treated between 2001 and 2025, the proportion of patients aged ≥ 80 years increased from 10% in 2001–2010 to 25% in 2021–2025 [3]. National registry data demonstrate similar demographic shifts with respect to colorectal surgery across Japan, with the proportion of patients aged ≥ 80 years increasing from 16.9% in 2011 to 21.5% in 2020 [4]. According to current guidelines, additional colectomy with lymph node dissection is considered after endoscopic resection (ER) when pathological features associated with a high risk of lymph node metastasis (LNM) are identified (Figure 1) [5]. These criteria—deep submucosal invasion, lymphovascular invasion, poor differentiation, and tumor budding—are well‐validated predictors of nodal involvement [6].

In real‐world practice, older patients frequently forgo additional surgery, even when they are at high risk [7]. In a population‐based analysis, increasing age was independently associated with the use of ER alone, rather than in combination with surgical resection. Compared with patients aged 66–69 years, the odds of avoiding surgery were progressively higher in those aged 75–79 years (odds ratio (OR) 1.59), 80–84 years (OR 2.79), and ≥ 85 years (OR 2.57), demonstrating a clear age‐dependent change in treatment selection [8]. In another study of patients with high‐risk T1 CRC, those who did not undergo additional surgical resection were significantly older than those who did [9].

This divergence between guideline‐based recommendations and their clinical implementation leads to the following question: Does additional surgery truly confer a meaningful survival benefit in older adults with limited life expectancy?

## 2 Methods

For this narrative review, we performed a structured literature search and selection process. KI and YK identified relevant articles through a manual search of PubMed up to January 2026. The search used combinations of the following keywords: “elderly,” “older adults,” “frailty,” “T1 CRC,” “early CRC,” “submucosal invasive CRC,” “endoscopic resection,” “endoscopic mucosal resection (EMR),” “endoscopic submucosal dissection (ESD),” “additional surgery,” “completion surgery,” “colectomy,” “lymph node dissection,” “surveillance,” “treatment strategy,” “LNM,” “recurrence,” “overall survival,” and “cancer‐specific survival.” All the authors contributed to the study selection. The titles and abstracts were screened, and the full texts of potentially relevant articles were reviewed. In the present review, older patients were defined as those aged ≥ 80 years.

## 3 Oncologic Rationale for Additional Surgery for Patients With T1 CRC

### 3.1 Objectives of Additional Bowel Resection

Additional bowel resection after ER for the treatment of T1 CRC has the aim of achieving oncologic clearance in patients with high‐risk pathologic features [5]. Clinicians can conceptualize the oncologic benefit of such additional surgery in three ways. First, surgery removes occult LNM that is frequently missed in preoperative CT/MRI nodal staging. For example, in a cohort of patients with early CRC, a short‐axis LN size criterion of 5 mm yielded low sensitivity (50%), despite moderate specificity (81.6%), in particular because metastatic nodes are often small in such patients [10]. Second, surgery reduces the risk of recurrence [11, 12]. Third, surgery improves the T1 CRC‐specific survival (CSS) of the patients [13]. The baseline risk of LNM in pT1 CRC is approximately 10% [6, 13, 14, 15, 16]; in high‐risk subgroups, the recurrence rate after local treatment alone ranges 6%–12%, and additional surgery reduces the incidence of recurrence to < 5%, as shown in most recent case series. Data collected over the long term have demonstrated favorable CSS for patients who undergo completion colectomy.

In addition, the magnitude of oncologic benefit may vary according to tumor location. Compared with T1 colon cancers, T1 rectal cancers tend to be larger and more frequently exhibit vascular invasion, although the rates of LNM appear similar between the two sites [17]. Furthermore, studies have shown that high‐risk T1 rectal cancers treated with endoscopic resection alone have substantially higher recurrence rates than comparable colon cancers [12]. In contrast, this difference is markedly attenuated among patients who undergo additional surgical resection with lymph node dissection.

### 3.2 Primary Surgery versus Stepwise Surgery After Endoscopic Resection

The timing of surgery remains a clinically important issue. Some clinicians question whether initially performing ER may compromise oncologic outcomes. The results of some studies have suggested that inadequate endoscopic treatment could increase malignant potential and the risk of metastatic disease [18, 19].

Recent retrospective cohort studies have compared a stepwise strategy of ER followed by additional surgery with primary surgery that includes lymph node resection [20, 21, 22, 23], and shown that the long‐term outcomes of the two approaches are comparable. A recent meta‐analysis has also demonstrated no significant difference in the long‐term oncologic outcomes of a stepwise strategy and primary surgery [24]. Although these studies were retrospective, their findings are consistent with the concept that ER does not impair the adequacy of oncologic treatment when surgeons perform timely additional resection when indicated. These findings imply that additional surgery after ER can achieve cure in patients with T1 CRC.

### 3.3 Effects of Additional Surgery on the Long‐Term Outcomes of the Patients

Beyond the theoretical validity of such additional surgery, the actual long‐term benefits must be considered. Large cohort studies have shown favorable outcomes for patients with high‐risk pathologic features who undergo additional bowel resection after ER [25], and these patients demonstrated low recurrence rates and favorable CSS. In contrast, studies have consistently shown higher recurrence rates in high‐risk patients who were managed by endoscopic resection alone. These findings suggest that additional surgery contributes not only to the removal of occult LNM, but also to sustained disease control. The survival benefit appears to be most pronounced in patients with established pathologic risk factors for LNM. Conversely, excellent long‐term outcomes have been demonstrated for those classified as being at low risk, according to current guidelines, without additional resection [13].

However, the magnitude of the oncologic benefit varies among patient populations. In a multicenter cohort study in Japan, the investigators evaluated high‐risk T1 cancers after endoscopic resection [25], and found lower‐than‐expected incidences of oncologic adverse events, even when additional surgery was not performed. In propensity score‐matched analyses, CSS remained excellent in the patients who did not undergo surgery, demonstrated by a small difference in the 5‐year CSS between the groups, with most deaths in the nonsurgical group having noncancer causes.

**FIGURE 2:**
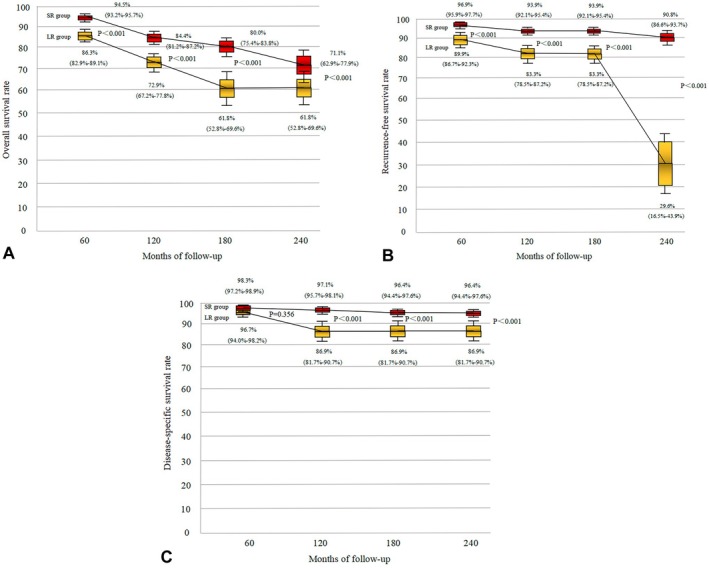
(A) Overall survival rates at different time nodes in the local resection (LR) and surgical resection (SR) groups. (B) Recurrence‐free survival rates at different time nodes in the LR and SR groups. (C) Disease‐specific survival rates at different time nodes in the LR and SR groups. Reproduced from Chen et al. in accordance with the publisher's permissions policy [26].

A recent meta‐analysis by Chen et al. evaluated the long‐term outcomes of patients with high‐risk T1 CRC who underwent local resection (LR; including ER and transanal excision) versus surgical resection (SR) (Figure 2) [26]. The analysis demonstrated that surgery was associated with improved disease‐specific survival (DSS) over prolonged follow‐up. The 5‐year DSS rates were similar between groups (96.7% vs. 98.3%), whereas more substantial differences emerged after 10 years (86.9% vs. 97.1%) and persisted at 20 years (86.9% vs. 96.4%). In contrast, the difference in overall survival (OS) remained relatively modest throughout follow‐up, with 5‐, 10‐, and 20‐year OS rates of 86.3% versus 94.5%, 72.9% versus 84.4%, and 61.8% versus 71.1% for local resection and surgical resection, respectively. These findings suggest that although additional surgery may improve cancer‐specific outcomes, its impact on OS is attenuated by competing causes of death. These findings indicate that the oncologic benefit of surgery accumulates gradually over time and should be interpreted in the context of competing mortality. Taken together, these findings support the oncologic rationale for additional surgery in patients with high‐risk T1 CRC. However, the translation of this oncologic benefit into net clinical benefit requires consideration of additional patient‐related factors beyond tumor biology.

## 4 Why the Magnitude of the Benefit Differs Among Older Adults

### 4.1 Competing Noncancer Mortality and the Attenuation of the Net Survival Benefit

Population‐based studies have repeatedly shown that many deaths of older patients with CRC have noncancer causes [27]. Indeed, noncancer mortality increases sharply with age and may exceed CRC‐specific mortality in older populations. As a result, even if additional surgery reduces the incidence of cancer‐related death, the OS benefit may appear small when competing noncancer deaths predominate [28]. This issue is particularly relevant for patients with T1 CRC, in whom the baseline incidence of cancer‐specific mortality remains low. When the underlying risk of cancer death is low, the absolute benefit of surgical intervention is highly sensitive to competing risks [29]. Therefore, the assessment of OS alone may not accurately reflect the true value of additional surgery in older patients, and clinicians should interpret survival outcomes within the context of competing mortality.

A similar paradigm shift has been reported for early gastric cancer [30]. In a multicenter study of 1065 patients aged ≥ 85 years who underwent ESD in Japan, 143 underwent noncurative resection, but only 11.2% underwent additional gastrectomy. Despite the omission of surgery for the majority, the 5‐year overall survival was comparable for the surgery and nonsurgery groups (63.1% vs. 65.2%; *p* = 0.891). For the patients who were managed without surgery, the 5‐year disease‐specific survival remained extremely high for the low‐ and intermediate‐risk eCura categories (100.0% and 97.1%, respectively), whereas the non‐gastric cancer‐specific survival was only 69.0%, indicating that there was substantial competing mortality. The eCura system is a validated risk stratification model that predicts LNM and the long‐term outcomes of the noncurative endoscopic resection of early gastric cancer, on the basis of pathologic factors [31]. Multivariable analysis identified high eCura risk (hazard ratio (HR) 2.91) and Charlson comorbidity index (CCI) ≥ 3 (HR 2.78), rather than the omission of surgery, as independent predictors of poor overall survival. These findings suggest that the uniform application of guideline‐recommended gastrectomy may not be appropriate for patients of very advanced age and that the omission of additional surgery can be a rational option for carefully selected patients.

### 4.2 Age‐Related Increases in Perioperative Risk

**FIGURE 3:**
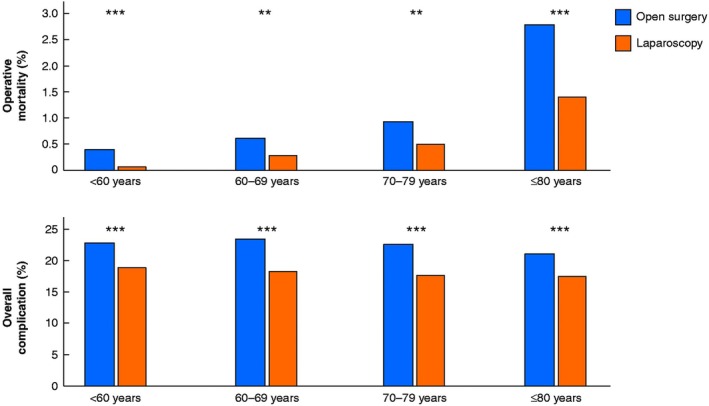
Comparison of operative mortality and overall postoperative complications between open surgery and laparoscopy in each age category ***p < 0.001, **p < 0.01. Reproduced from Seishima et al. in accordance with the publisher's permissions policy [33].

Population‐based analyses have shown that the incidences of postoperative complications and short‐term mortality increase steadily with age after colorectal surgery [32]. In a nationwide Japanese study, perioperative mortality in patients aged ≥ 80 years reached 2.2% (95% confidence interval [CI] 1.9%–2.6%), compared with 0.2% (95% CI 0.15%–0.31%) in those of < 60 years [33]. Figure 3 in the publication demonstrated a consistent age‐dependent increase in mortality in association with both open surgery and laparoscopy. For patients aged ≥ 80 years, the incidence of perioperative mortality was 2.8% after open surgery and 1.5% after laparoscopy. In contrast, the incidence of mortality in patients of < 60 years remained below 0.5%, regardless of the surgical approach used. Similarly, Omichi et al. reported that the odds of 30‐day postoperative mortality was much higher in patients aged ≥ 80 years than in those of < 60 years. The odds ratio was 1.61–2.25 for right hemicolectomy and 3.73–8.25 for low anterior resection [34]. However, most available data have been derived from studies that included patients with CRC across all stages. Therefore, these estimates may not accurately reflect the short‐term surgical risk among patients with T1 CRC, for whom direct evidence remains limited.

## 5 Current Limitations and Unresolved Issues

Despite having accumulated observational evidence, several important knowledge limitations remain in this field. First, most of the available studies were retrospective and subject to selection bias because the patients who underwent additional surgery were in general fitter than those who were managed nonsurgically [8]. In addition, frailty indices and competing mortality risks were inconsistently measured and rarely integrated into multivariable models. Second, although meta‐analyses have suggested that the survival advantage of surgery emerges after prolonged follow‐up of > 10 years, few studies have formally quantified the time‐to‐benefit relative to the remaining life expectancy for patients aged ≥ 80 years [26]. Third, the current guideline criteria rely primarily on pathologic risk factors and do not incorporate a structured geriatric assessment [5]. As a result, clinicians lack a validated framework for the estimation of the individualized net clinical benefit that balances oncologic gain with perioperative risk and competing mortality. These gaps in knowledge underscore the need for the development of integrative models that are capable of supporting personalized decision‐making in older adults with pT1 CRC.

Another unresolved issue is whether selected patients may benefit from organ‐preserving alternatives to additional surgery. Recently, the TESAR trial evaluated adjuvant chemoradiotherapy after local excision of high‐risk pT1 and low‐risk pT2 rectal cancer as an organ‐preserving alternative to completion surgery [35]. Although non‐inferiority for locoregional recurrence was not formally demonstrated, adjuvant chemoradiotherapy was associated with lower treatment‐related morbidity and stoma formation. These findings suggest that nonoperative treatment strategies may provide a potential alternative for carefully selected rectal cancer patients who face substantial surgical risk, although further validation is required.

## 6 Artificial Intelligence (AI) as a Decision Integrator

### 6.1 Toward an Individualized Assessment of the Net Benefit

Decision‐making after endoscopic resection for the treatment of pT1 CRC in older adults should move beyond the consideration of tumor pathology alone and incorporate a structured geriatric assessment [36]. Furthermore, objective vulnerability metrics should be systematically integrated into the evaluation of surgical indications.

Several validated instruments are available for this purpose. The CCI quantifies competing mortality risk and has consistently predicted the OS of older gastrointestinal cancer cohorts [37, 38]. The G8 screening tool enables the rapid identification of frailty and predicts postoperative complications and noncancer‐related mortality [39, 40]. The ASA physical status classification provides an estimate of perioperative risk, and imaging‐based markers such as sarcopenia, assessed using a CT‐derived skeletal muscle index, reflect diminished physiologic reserve and are independently associated with postoperative morbidity and poorer long‐term survival [41, 42, 43].

**FIGURE 4:**
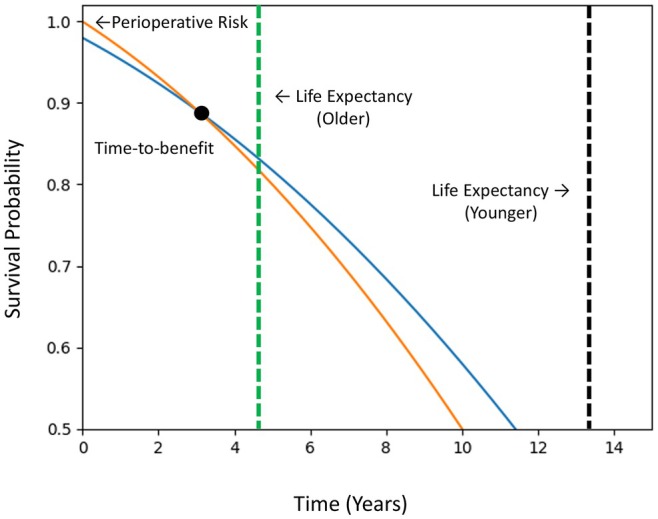
Conceptual model of net clinical benefit over time in older adults with pT1 colorectal cancer. The blue curve represents survival after additional surgery, incorporating early perioperative risk followed by reduced recurrence‐related mortality. The orange curve represents survival without additional surgery, characterized by gradual recurrence‐related decline without immediate surgical risk. In some older adults, competing mortality and frailty may limit the clinical relevance of the delayed survival benefit associated with surgery. This figure is a conceptual model created by the authors and is not derived from original clinical data.

Incorporating these parameters allows clinicians to estimate not only oncologic risk, but also perioperative vulnerability and the risk of competing mortality. Such a multidimensional framework shifts the focus from “Is LNM possible?” to “Will surgery meaningfully improve this patient's long‐term outcome?” This net benefit perspective is particularly important for patients of very advanced age, for whom small oncologic gains may be offset by the immediate surgical risks and their limited remaining lifespan (Figure 4).

### 6.2 Potential Role of AI in Decision Integration

AI offers an opportunity to operationalize this multidimensional assessment. AI has demonstrated promising performance in predicting LNM compared with existing guideline‐based risk stratification systems and conventional multivariable logistic regression models in patients with T1 CRC [44]. One potential advantage of AI is its ability to integrate a large number of heterogeneous variables, including pathological features, tumor characteristics, clinical factors, and patient‐related parameters, while capturing complex nonlinear relationships among these variables.

Rather than predicting LNM alone, next‐generation decision‐support AI should integrate pathologic risk factors, frailty metrics (CCI, G8, and sarcopenia indices), the risk of competing mortality, and the risk of perioperative complications into a unified net clinical benefit model [45]. Such models could be used to estimate the individualized absolute survival gain after 5, 10, and 15 years, while simultaneously quantifying the short‐term surgical harm. By presenting predicted survival curves for both surgical and nonsurgical strategies, AI systems may facilitate transparent shared decision‐making for physicians and older patients. However, treatment decisions in older adults are influenced not only by clinical risk but also by patient values, personal preferences, family circumstances, and perspectives on quality of life and longevity, factors that may not be fully captured by quantitative models. Therefore, AI should be viewed as a decision‐support tool that complements, rather than replaces, individualized shared decision‐making. A calibrated AI framework that balances oncologic control against geriatric vulnerability would represent a paradigm shift from guideline‐driven uniformity toward truly personalized treatment selection.

## 7 Conclusions

In older patients with pT1 CRC, the benefit of additional surgery after endoscopic resection depends on the balance between the oncologic gain and the risk of competing mortality. Although surgery reduces the risk of recurrence for patients with high‐risk disease, its net survival advantage may not translate into a meaningful extension of survival in many older patients who have frailty, limited life expectancy, and/or a high perioperative risk. Therefore, treatment decisions should be based on more than pathology alone and incorporate individualized assessment of geriatric vulnerability.

## Author Contributions

K.I., Y.K., and M.M.: conceptualization. K.I., S.K., Y.K., T.O., Y.M., T.H., K.G.Y., H.M., and M.M.: data (articles) interpretation. K.I.: writing – original draft. S.K., Y.K., T.O., Y.M., T.H., K.G.Y., H.M., and M.M.: writing – review and editing. S.K., K.G.Y., H.M., and M.M.: supervision.

## Funding

The authors have nothing to report.

## Ethics Statement

The authors have nothing to report.

## Consent

The authors have nothing to report.

## Conflicts of Interest

M.M. is an Associate Editor of Digestive Endoscopy. Y.M. is an Associate Editor of DEN Open. The other authors declare no conflicts of interest.
